# Durable Response after Repeat Cytoreductive Surgery (CRS) and Hyperthermic Intraperitoneal Chemotherapy (HIPEC) in a Patient with Extensive Mucinous Adenocarcinoma of the Appendix

**DOI:** 10.3390/diseases11020060

**Published:** 2023-04-06

**Authors:** Dalia Kaakour, Garrett Ward, Maheswari Senthil, Farshid Dayyani

**Affiliations:** 1 Department of Medicine, Division of Hematology and Oncology, University of California Irvine, Orange, CA 92868, USA; 2 Department of Radiological Sciences, Division of Abdominal Imaging, University of California Irvine, Orange, CA 92868, USA; 3 Department of Surgery, Division of Surgical Oncology, University of California Irvine, Orange, CA 92868, USA

**Keywords:** mucinous adenocarcinoma of the appendix, peritoneal carcinomatosis, cytoreduction and HIPEC

## Abstract

Mucinous adenocarcinoma of the appendix is a rare form of lower gastrointestinal (GI) tract cancer. These cancers have a high tendency to progress towards peritoneal metastasis and their response to systemic treatment is typically low. Together, cytoreductive surgery (CRS) and hyperthermic intraperitoneal chemotherapy (HIPEC) have become an established form of therapy used to prolong the survival of patients with this disease. Repeat CRS and HIPEC have been shown to be feasible in selected patients with GI peritoneal carcinomatosis (PC), among which those with appendix cancer receive the greatest benefit. The peritoneal cancer index (PCI) and completeness of cytoreduction have been shown to be important predictors of outcomes. However, repeat cytoreduction in patients with a high-volume peritoneal tumor burden (peritoneal cancer index (PCI) > 30) is not typically performed due to concerns regarding morbidity and mortality. Herein, we describe a case of repeat CRS and HIPEC for extensive appendiceal mucinous peritoneal carcinomatosis after initial incomplete cytoreduction and durable remission of 28 months without adjuvant chemotherapy. In appendiceal mucinous cancers, repeat CRS can achieve a durable response despite an initial failed CRS and high-volume disease.

## 1. Introduction

Mucinous adenocarcinoma (MAC) of the appendix is a relatively uncommon form of GI malignancy and accounts for <0.5% of all gastrointestinal neoplasms [1]. The incidence of MAC of the appendix is rapidly increasing [2], and patients often present with a more advanced stage, typically due to metastasis in the peritoneal cavity. The extent of surgical resection for localized appendiceal mucinous adenocarcinoma is slightly controversial. However, generally speaking, an appendectomy alone may be adequate for low-grade disease if negative margins are achieved [3,4]. Right hemicolectomy is considered the standard-of-care therapy for high-grade disease due to the risk of regional lymph node spread, which is reported in 17–72% of cases [4]. Usually, peritoneal metastases of MAC remain isolated to the peritoneal cavity, rendering these patients appropriate candidates for aggressive locoregional therapies [5]. The combination of cytoreductive surgery (CRS) to remove all visible disease followed by hyperthermic intraperitoneal chemoperfusion (HIPEC) to eliminate microscopic disease has become the standard treatment [5]. Regarding the systemic treatment of MACs with peritoneal carcinomatosis (PC), no association has been found between chemotherapy and overall survival in patients with low-grade mucinous appendiceal adenocarcinoma, indicating a poor response to systemic treatment [3,4]. On the other hand, it is recommended that patients with high-grade disease exhibiting PC undergo preoperative systemic chemotherapy in addition to CRS and HIPEC, as this has shown advantageous results in terms of progression-free survival [6].

The survival benefit of repeat CRS and HIPEC in appropriately selected patients with appendix PC has been reported [5], for which the peritoneal cancer index (PCI) and completeness of cytoreduction have proven to be important predictors of outcomes. However, repeat cytoreduction in patients with a high-volume peritoneal tumor burden (PCI > 30) is not typically performed due to concerns regarding morbidity and mortality. Herein, we describe a case of repeat CRS and HIPEC for extensive appendiceal mucinous peritoneal carcinomatosis after initial incomplete cytoreduction and durable remission of 28 months without adjuvant chemotherapy.

## 2. Case

**Figure 1:**
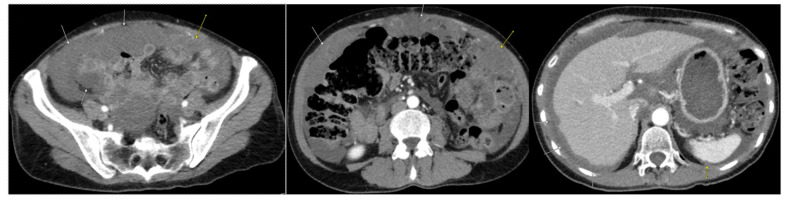
Initial CT scan of abdomen/pelvis completed at time of diagnosis. Arrows indicate diffuse mucinous peritoneal metastases throughout the abdomen and pelvis.

A 53-year-old female with a medical history of cesarean section and bilateral tubal ligation presented to her primary care physician with a six-month history of abdominal distention, bloating, early satiety, and unintentional weight loss amounting to 25 pounds. At this point, a CT scan of her abdomen and pelvis revealed ascites, peritoneal implants, and bilateral adnexal masses (Figure 1). The observed tumor markers were as follows: CA 19-9—1398 units per milliliter (U/mL); CA-125—118 U/mL; and CEA—55.4 micrograms per liter (µg/L). Cytology from her paracentesis revealed adenocarcinoma that immunohistochemically favored an upper gastrointestinal versus a pancreaticobiliary origin. However, EGD, EUS, and colonoscopy revealed no masses in the stomach, duodenum, pancreas, liver, or colon, hence indicating a possible appendiceal origin.

A diagnostic laparoscopy with an appendectomy was performed. The findings indicated extensive mucinous peritoneal carcinomatosis. A pathologic review showed invasive mucinous adenocarcinoma of the appendix, which was 3 cm, predominantly well-differentiated, of a low grade, and possessed a focal component with moderate differentiation of an intermediate grade. Molecular studies showed that the patient’s microsatellite status was microsatellite-stable (MSS) and that her tumor mutational burden (TMB) was low at 4 Muts/Mb. Additionally, RAS G12V and GNAS R201C were detected.

**Figure 2:**
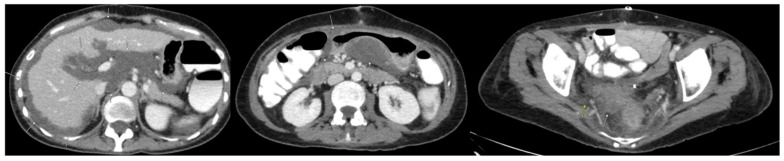
CT scan of the abdomen/pelvis conducted after first CRS/HIPEC. Arrows indicate residual mucinous peritoneal metastases and demonstrate interval reduction in tumor burden.

**Figure 3:**
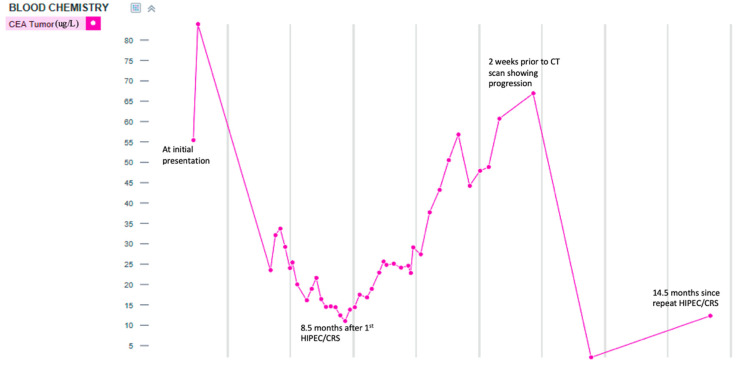
Trend in CEA level over time.

The patient was considered a candidate for CRS/HIPEC; however, intraoperatively, the surgeons felt that a complete cytoreduction was not feasible due to extensive tumors in the upper abdomen, porta, and small bowel. Hence, an incomplete resection consisting of a right colectomy, total abdominal hysterectomy, bilateral oophorectomy, and omentectomy was performed (Figure 2). Even though significant gross disease was still present, the patient received oxaliplatin HIPEC. Since the patient still had active disease and was deemed an unsuitable candidate for surgical cytoreduction, a course of systemic chemotherapy was started. Over the course of 24 months post-incomplete CRS, the patient received six cycles of FOLOFOX, FOLFIRI, and FOLFIRI with Bevacizumab and Lonsurf Trifluridine/Tipiracil (TAS-102) with down-trending CEA (Figure 3); however, there was no significant imaging response.

**Figure 4:**
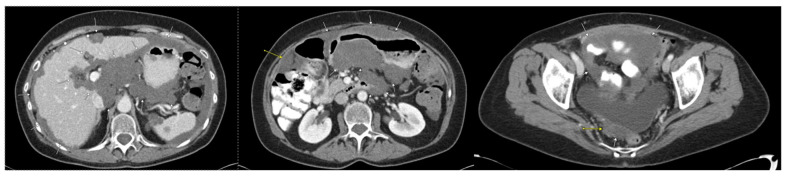
First CT scan of abdomen/pelvis after initial CRS/HIPEC to show disease progression. Arrows indicate diffuse and enlarged mucinous peritoneal metastases and demonstrate interval increase in tumor burden.

Repeat CT after four lines of systemic chemotherapy showed no intrathoracic metastasis and a diffuse, increased volume of low-density peritoneal disease that was more extensive than that observed in prior imaging, with a clear progression of disease in the pelvis and upper abdomen (Figure 4). At this point, the patient was referred to our program for cytoreduction. Given the patient’s young age, the fact that nearly two years had passed since the original cytoreduction, and the patient’s excellent performance status, we decided to proceed with repeat cytoreduction despite the presence of extensive peritoneal disease.

The important intraoperative findings obtained included a dense mucinous tumor engulfing the entire upper abdominal contents, including the porta hepatis, retro-caudate space, lesser sac, bilateral diaphragms, spleen, stomach, and pancreas. There was also disease involving the colon, rectum, and pelvis. The small bowel had a diffuse mucinous tumor; however, it was not deeply invasive. The PCI was 37. Due to the extent of disease, the patient required a composite resection of a large conglomerate of mucinous tumor mass (>45 cm), including distal near total stomach, distal subtotal pancreas, and spleen resections. An extensive bilateral diaphragmatic peritonectomy with a resection of portions of the diaphragm on both sides was performed. The patient also required a complete parietal peritonectomy and total proctocolectomy with a pelvic peritonectomy. The small bowel disease was managed with a mesenteric peritonectomy, small bowel resections, and serosal resections, with residual small bowel of over 200 cm. At this point, after 19 h of surgery, the patient became hemodynamically labile; hence, the abdomen was temporarily closed, and the patient was transferred to the ICU for continued resuscitation. There were still tumors along both sides of the retrohepatic cava, and there was a bulky tumor encompassing the porta circumferentially and a retro-caudate tumor extending to the right crus of the diaphragm. The patient was brought back six hours later for the completion of the cytoreduction, which included the removal of the bulky porta hepatis tumor and peri vena caval tumors and Mitomycin HIPEC for 90 min, followed by a gastrojejunostomy and the creation of an end ileostomy. The total duration of cytoreduction was 26 h over two consecutive days.

**Figure 5:**
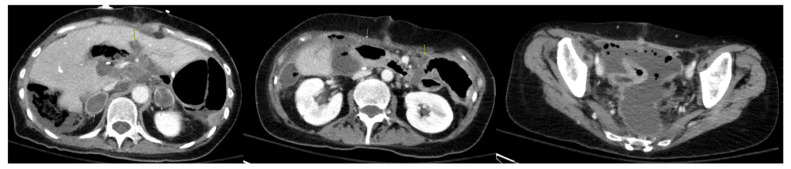
CT of the abdomen/pelvis after second CRS/HIPEC. Arrows indicate sites of residual postoperative collections.

The patient recovered from this extensive surgery without any acute surgical complications. She required postoperative parenteral nutrition due to failure to thrive and malnutrition. Several MRI scans of the patient’s peritoneum showed post-operative changes consistent with fluid collection. The follow-up CT scans over 5 months (Figure 5), 9 months, and 16 months all appeared to be stable.

At the time of this report, the patient has been off any systemic chemotherapy for a total duration of 30 months. Although the patient’s overall quality of life (QOL) and functional status declined for the first six months postoperatively, she regained her functional status and QOL to a near baseline status by 14 months after the second CRS/HIPEC procedure. To date, her CT scans remain stable, with negative (0.00) results regarding molecular residual disease testing with plasma ctDNA after 28 months.

## 3. Discussion

Appendiceal adenocarcinomas can be classified into five primary groups: neuroendocrine neoplasms, mucinous adenocarcinomas, goblet cell tumors, colonic-type adenocarcinomas, and signet ring cell adenocarcinomas [7]. Histologically, invasive mucinous adenocarcinomas of the appendix have pools of mucin infiltrating the wall of the appendix containing cytologically malignant glandular epithelia, which rupture and metastasize in the peritoneum [8,9].

The combination of cytoreductive surgery and HIPEC has been used as an aggressive treatment approach that results in better long-term survival [10,11,12]. The long-term survival rates at 5 and 10 years are approximately 71.9% and 54.5% in CRS and HIPEC patients, which can be compared to rates of approximately 53% and 32% in patients that underwent debulking [9,12]. Although CRS and HIPEC have been effective in terms of improving long-term survival, recurrence will eventually occur in up to 70% of patients, especially those presenting with voluminous disease (PCI > 20). If left untreated, the majority of these patients will succumb to bowel obstruction. In this population, the combined use of repeat CRS and HIPEC has been offered to up to 20% of patients with low-grade disease [13].

Several studies have examined the joint use of repeat CRS and perioperative intraperitoneal chemotherapy (PIC) in patients with recurrent disease. In one of the earliest series conducted by Esquivel and Sugarbaker, of the initial 321 appendix PC patients that underwent CRS over a 12-year-period, repeat CRS was performed for 67 patients (21%). The 5-year survival of patients who underwent repeat CRS was 84% if complete cytoreduction was achieved compared to 44% in a group that had incomplete cytoreduction, thus highlighting the importance of complete cytoreduction [14]. Multiple studies have shown increased survival after repeat CRS and HIPEC in patients with both low-grade and high-grade disease, corresponding to 5-year survival values of 81.3% vs. 46.3% (*p* < 0.001) and 50% vs. 12% (*p* = 0.02) [15]. The joint use of repeat CRS and HIPEC is technically feasible and offers demonstrated benefits in long-term survival. We acknowledge that there is a significant decrease in QOL during the immediate post-operative period following repeat CRS; however, since the majority of patients will regain their baseline functional status and have an acceptable QOL, repeat CRS should be discussed with patients to allow for shared decision making.

To the best of our knowledge, there are no studies comparing the survival of patients with mucinous adenocarcinoma of the appendix who underwent repeat CRS and HIPEC with and without receiving systemic chemotherapy postoperatively.

Additionally, the molecular profiling of patients with mucinous cancers more often shows microsatellite instability (MSI) when compared with adenocarcinoma. It is also common to find BRAF mutations and an increased rate of KRAS and PIK3CA mutations in patients with mucinous cancer [16]. The patient in this case did not have MSI but did have a KRAS mutation, namely, KRAS G12V. She initially underwent incomplete cytoreduction and HIPEC followed by systemic chemotherapy, which was switched from FOLFOX to FOLFIRI as she had persistent peritoneal disease and worsening neuropathy. Her treatment was then switched to TAS-102, and her disease remained stable with respect to her CT scans and CEA tumor markers. Ultimately, her disease progressed, requiring repeat CRS and HIPEC. She did not receive adjuvant chemotherapy after this combined treatment and currently maintains stable values with respect to MRI imaging, thereby bringing to question the value of post-operative adjuvant chemotherapy in patients with mucinous adenocarcinoma of the appendix with peritoneal disease following repeat CRS and HIPEC. This is an area requiring further investigation with respect to the use and benefit of adjuvant chemotherapy and whether it has any effect on mortality or provides any survival benefits.

Lastly, it is important to consider a patient’s post-operative quality of life after undergoing CRS and HIPEC. Numerous studies have examined the global, physical, functional, social, and emotional QOL of patients receiving CRS and HIPEC [17,18,19,20,21,22,23,24]. Careful patient selection is critical, as prognostication is of the utmost importance since the completeness of cytoreduction is a leading factor in post-operative patient outcomes [25,26]. Not surprisingly, short-term impairment of QOL is expected in the early post-operative period, and QOL improvement can be seen at variable intervals, with one study suggesting that QOL improves as early as 3 months post-operation [18]. However, the majority of studies on this topic suggest that QOL improvement requires 6–12 months [20,21]. There are a number of post-operative programs and interventions that have been shown to improve QOL, including patient education programs, psychological counseling, cognitive therapies, and exercise programs, which should be considered [27,28,29].

## 4. Conclusions

The patient related herein demonstrates that it is possible to have a durable response without adjuvant chemotherapy after repeat CRS and HIPEC. Further, it is important to note that the morbidity and mortality associated with CRS and HIPEC are significant [30]. Patients must be carefully selected, and further research into the patient-specific and operative factors that influence the outcomes as well as post-operative complications is needed [31]. Lastly, we must stress the surgeon-dependent nature of CRS and HIPEC outcomes, as there are both technical learning curves and learning curves associated with patient selection [32,33,34]. Optimal CRS is critical and repeat CRS should be considered as a treatment option regardless of whether an initial attempt has failed.

## Data Availability

Not applicable.

## References

[1] Chen W., Ye J.W., Tan X.P., Peng X., Zhang Y., Liang J.L., Huang M.J. (2020). A case report of appendix mucinous adenocarcinoma that recurred after additional surgery and a brief literature review. BMC Surg..

[2] Xie G.D., Liu Y.R., Jiang Y.Z., Shao Z.M. (2018). Epidemiology and survival outcomes of mucinous adenocarcinomas: A SEER population-based study. Sci. Rep..

[3] Lu P., Fields A.C., Meyerhardt J.A., Davids J.S., Shabat G., Bleday R., Goldberg J.E., Nash G.M., Melnitchouk N. (2019). Systemic chemotherapy and survival in patients with metastatic low-grade appendiceal mucinous adenocarcinoma. J. Surg. Oncol..

[4] Davison J.M., Choudry H.A., Pingpank J.F., Ahrendt S.A., Holtzman M.P., Zureikat A.H., Zeh H.J., Ramalingam L., Zhu B., Nikiforova M. (2014). Clinicopathologic and molecular analysis of disseminated appendiceal mucinous neoplasms: Identification of factors predicting survival and proposed criteria for a three-tiered assessment of tumor grade. Mod. Pathol..

[5] Choudry H.A., Pai R.K. (2018). Management of Mucinous Appendiceal Tumors. Ann. Surg. Oncol..

[6] Blackham A.U., Swett K., Eng C., Sirintrapun J., Bergman S., Geisinger K.R., Votanopoulos K., Stewart J.H., Shen P., Levine E.A. (2014). Perioperative systemic chemotherapy for appendiceal mucinous carcinoma peritonei treated with cytoreductive surgery and hyperthermic intraperitoneal chemotherapy. J. Surg. Oncol..

[7] Hechtman J., Bahary N. (2021). Current Management of Appendiceal Neoplasms. Am. Soc. Clin. Oncol. Educ. Book.

[8] Misdraji J. (2015). Mucinous epithelial neoplasms of the appendix and pseudomyxoma peritonei. Mod. Pathol..

[9] Gonzalez-Moreno S., Sugarbaker P.H. (2004). Right hemicolectomy does not confer a survival advantage in patients with mucinous carcinoma of the appendix and peritoneal seeding. Br. J. Surg..

[10] Smeenk R.M., Verwaal V.J., Antonini N., Zoetmulder F.A. (2007). Survival analysis of pseudomyxoma peritonei patients treated by cytoreductive surgery and hyperthermic intraperitoneal chemotherapy. Ann. Surg..

[11] Elias D., Gilly F., Quenet F., Bereder J.M., Sidéris L., Mansvelt B., Lorimier G., Glehen O., Association Française de Chirurgie (2010). Pseudomyxoma peritonei: A French multicentric study of 301 patients treated with cytoreductive surgery and intraperitoneal chemotherapy. Eur. J. Surg. Oncol..

[12] Gough D.B., Donohue J.H., Schutt A.J., Gonchoroff N., Goellner J.R., Wilson T.O., Naessens J.M., O’Brien P.C., Van Heerden J.A. (1994). Pseudomyxoma peritonei. Long-term patient survival with an aggressive regional approach. Ann. Surg..

[13] Votanopoulos K.I. (2022). Repeat CRS/HIPEC: It Comes Down to Tumor Biology and Ability to Achieve a Complete CRS. Ann. Surg. Oncol..

[14] Esquivel J., Sugarbaker P.H. (2001). Second-look surgery in patients with peritoneal dissemination from appendiceal malignancy: Analysis of prognostic factors in 98 patients. Ann. Surg..

[15] Lopez-Ramirez F., Gushchin V., Sittig M., King M.C., Baron E., Nikiforchin A., Nieroda C., Sardi A. (2022). Iterative Cytoreduction and Hyperthermic Intraperitoneal Chemotherapy for Recurrent Mucinous Adenocarcinoma of the Appendix. Ann. Surg. Oncol..

[16] Hugen N., Brown G., Glynne-Jones R., de Wilt J.H., Nagtegaal I.D. (2016). Advances in the care of patients with mucinous colorectal cancer. Nat. Rev. Clin. Oncol..

[17] Passot G., Bakrin N., Roux A.S., Vaudoyer D., Gilly F.N., Glehen O., Cotte E. (2014). Quality of life after cytoreductive surgery plus hyperthermic intraperitoneal chemotherapy: A prospective study of 216 patients. Eur. J. Surg. Oncol..

[18] McQuellon R.P., Loggie B.W., Fleming R.A., Russell G.B., Lehman A.B., Rambo T.D. (2001). Quality of life after intraperitoneal hyperthermic chemotherapy (IPHC) for peritoneal carcinomatosis. Eur. J. Surg. Oncol..

[19] Macrì A., Maugeri I., Trimarchi G., Caminiti R., Saffioti M.C., Incardona S., Sinardi A., Irato S., Altavilla G., Adamo V. (2009). Evaluation of quality of life of patients submitted to cytoreductive surgery and hyperthermic intraperitoneal chemotherapy for peritoneal carcinosis of gastrointestinal and ovarian origin and identification of factors influencing outcome. In Vivo.

[20] Alves S., Mohamed F., Yadegarfar G., Youssef H., Moran B.J. (2010). Prospective longitudinal study of quality of life following cytoreductive surgery and intraperitoneal chemotherapy for pseudomyxoma peritonei. Eur. J. Surg. Oncol..

[21] Lim C., Tordjmann D., Gornet J.M., Nemeth J., Valleur P., Pocard M. (2010). Prospective study of quality of life after cytoreductive surgery and hyperthermic intraperitoneal chemotherapy using oxaliplatin for peritoneal carcinomatosis. Bull. Cancer.

[22] Chia C.S., Tan W.J., Wong J.S., Tan G.C., Lim C., Wang W., Tham C.K., Soo K.C., Teo M.C. (2014). Quality of life in patients with peritoneal surface malignancies after cytoreductive surgery and hyperthermic intraperitoneal chemotherapy. Eur. J. Surg. Oncol..

[23] Tan W.J., Wong J.F., Chia C.S., Tan G.H., Soo K.C., Teo M.C. (2013). Quality of life after cytoreductive surgery and hyperthermic intraperitoneal chemotherapy: An Asian perspective. Ann. Surg. Oncol..

[24] Tun S., Eng O.S., Malec M. (2020). Health-Related Quality of Life After Cytoreductive Surgery/HIPEC for Mucinous Appendiceal Cancer: Results of a Multicenter Randomized Trial Comparing Oxaliplatin and Mitomycin. Ann. Surg. Oncol..

[25] Glockzin G., Schlitt H.J., Piso P. (2009). Peritoneal carcinomatosis: Patients selection, perioperative complications and quality of life related to cytoreductive surgery and hyperthermic intraperitoneal chemotherapy. World J. Surg. Oncol..

[26] Ashvin R., Nikhilesh J. (2016). Preoperative Preparation and Patient Selection for Cytoreductive Surgery and HIPEC. Indian J. Surg. Oncol..

[27] Naumann F., Martin E., Philpott M., Smith C., Groff D., Battaglini C. (2012). Can counseling add value to an exercise intervention for improving quality of life in breast cancer survivors? A feasibility study. J. Support. Oncol..

[28] Cheng K.K., Lim Y.T., Koh Z.M., San Tam W.W. (2017). Home-based multidimensional survivorship programmes for breast cancer survivors. Cochrane Database Syst. Rev..

[29] Gao Q., Li H., Zou Y., Hou B., Liu L. (2020). Effectiveness of a comprehensive post-operative health education program in improving quality of life after gastric cancer surgery. Ann. Palliat. Med..

[30] Wu Z., Li Z., Ji J. (2016). Morbidity and mortality of cytoreductive surgery with hyperthermic intraperitoneal chemotherapy in advanced gastric cancer. Transl. Gastroenterol. Hepatol..

[31] Newton A.D., Bartlett E.K., Karakousis G.C. (2016). Cytoreductive surgery and hyperthermic intraperitoneal chemotherapy: A review of factors contributing to morbidity and mortality. J. Gastrointest. Oncol..

[32] Smeenk R.M., Verwaal V.J., Zoetmulder F.A. (2007). Learning curve of combined modality treatment in peritoneal surface disease. Br. J. Surg..

[33] Turrini O., Lambaudie E., Faucher M., Viret F., Blache J.L., Houvenaeghel G., Delpero J.R. (2012). Initial experience with hyperthermic intraperitoneal chemotherapy. Arch. Surg..

[34] Chua T.C., Yan T.D., Smigielski M.E., Zhu K.J., Ng K.M., Zhao J., Morris D.L. (2009). Long-term survival in patients with pseudomyxoma peritonei treated with cytoreductive surgery and perioperative intraperitoneal chemotherapy: 10 years of experience from a single institution. Ann. Surg. Oncol..

